# Comparison of the Rate of Posterior Capsule Opacification Following Combined Treatment With Topical Dexamethasone 0.1% Plus Ketorolac 0.5% Eye Drops Versus Dexamethasone 0.1% Alone: A Two-Year, Randomized Clinical Investigation

**DOI:** 10.7759/cureus.37223

**Published:** 2023-04-06

**Authors:** Cem Evereklioglu, Hatice Arda, Hidayet Sener, Osman A Polat, Koray Gumus, Fatih Horozoglu

**Affiliations:** 1 Department of Ophthalmology, Division of Cataract and Refractive Surgery, Erciyes University Medical Faculty, Kayseri, TUR; 2 Department of Ophthalmology, Memorial Ankara Hospital, Ankara, TUR

**Keywords:** posterior capsule opacification, phacoemulsification, non-steroidal anti-inflammatory drug, ketorolac, dexamethasone, cataract

## Abstract

Background and aim

The use of non-steroidal anti-inflammatory drugs in animals decreases the incidence of posterior capsular opacification (PCO) following cataract surgery. We evaluated the rate of PCO in patients with cataract surgery and foldable “in the bag” posterior chamber intraocular lens (PC-IOL) implantation treated with combined dexamethasone 0.1% *plus *ketorolac tromethamine 0.5% *versus *dexamethasone 0.1% alone.

Materials and methods

A total of 114 eyes of 101 patients underwent uneventful corneal small-incision phacoemulsification with primary implantation of a foldable acrylic PC-IOL (AcrySof^®^, Alcon, Fort Worth, USA). Postoperatively for four weeks, group 1 eyes were treated with dexamethasone 0.1% *plus *ketorolac tromethamine 0.5% ophthalmic solutions four times daily for each whereas group 2 eyes were treated with dexamethasone 0.1% alone. Other regiments were the same for each group. Patients were evaluated between one- and four-year following surgery. The frequency and timing of severe PCO following surgery that needed Nd:YAG laser posterior capsulotomy were recorded and evaluated.

Results

The mean (SEM) age of group 1 (*n* = 54) and group 2 (*n* = 60) at operation was similar (62.8 ± 2.2 *vs*. 60.6 ± 1.7 years, respectively). Eighty-eight patients had unilateral cataract and 13 cases had bilateral disease. Overall, the mean follow-up duration was 24.7 months postoperatively (range, 15-48). Clinically significant PCO that finally needed Nd:YAG laser application developed in two eyes (3.7%) in group 1 and in four eyes (6.6%) in group 2, and the difference was not statistically significant (p>0.05). The mean month at capsulotomy was 26.5 in group 1 and 24.3 months in group 2 eyes (p>0.05).

Conclusions

Topical instillation of ketorolac ophthalmic solution in the immediate period after phacoemulsification and PC-IOL implantation did not seem to influence the incidence of PCO formation two years after cataract surgery.

## Introduction

Cataract surgery is the most common surgical procedure in the world and the most frequent long-term postoperative complication is posterior capsule opacification (PCO) which reaches 50% in many investigations, many of which require Nd:YAG laser capsulotomy [1]. Although modified surgical techniques including cortical cleanup with improvements in intraocular lens (IOL) design, size, shape, and chemical composition have been developed to reduce PCO formation, this problem is by no means resolved and after-cataract formation still develops as a result of re-proliferation, migration, and metaplasia of residual lens epithelial cells (LECs) from the anterior subcapsular epithelium and the remnants of lenticular fibers, which transform into fibroblasts and myofibroblasts along the posterior lens capsule [2-4]. Therefore, some novel modifications in our surgeries and pre- and postoperative medications are promptly required for the prevention of PCO in cataract surgeries.

Both in vivo and in vitro studies [5,6] including ours [7] have demonstrated that numerous pharmacological agents with antimitotic, antifibroblastic, or antineoplastic activities as well as molecular biology and gene therapy may potentially be effective for the prevention or control of PCO following cataract surgery, which is related to their irreversibly damaging and inhibitory effects on the LECs or the fibers. Indeed, laboratory studies on human LECs have demonstrated that non-steroidal anti-inflammatory drugs (NSAIDs) decrease the frequency of PCO following cataract surgery by the inhibition of cell division, metaplasia, and collagen synthesis of LECs that was supported by animal studies [8]. Moreover, NSAID-coated IOLs resulted in significantly less PCO rates when compared to contralateral control eyes with uncoated IOLs [9].

We demonstrated that postoperative long-term use of topical ketorolac drops in pediatric cataract patients with intact posterior capsules prevented PCO formation [10]. Similarly, hydrodissection using diclofenac sodium prevented PCO up to two years postoperatively [11]. Moreover, a recent in vitro and in vivo study has demonstrated that IOLs loaded with ketorolac reduce the tendency for adhesion of LECs, and no PCO signs were encountered [12]. Therefore, the aim of the present investigation was to compare the efficacy of topical dexamethasone 0.1% plus ketorolac tromethamine 0.5% eye drops versus dexamethasone alone regarding the prevention of PCO two years after phacoemulsification with primary implantation of a posterior chamber IOL (PC-IOL).

## Materials and methods

Patients admitted for elective cataract surgery with the implantation of an IOL were recruited from the Department of Ophthalmology, Division of Cataract and Refractive Surgery, Erciyes University Medical Faculty, Kayseri, Turkey, for this investigation. The study conformed to the tenets of the Declaration of Helsinki, and the study was approved by the Institutional Review Board (Approval number: 2023/37). Since most review committees no longer permit placebo-controlled studies following phacoemulsification in humans, the present study used dexamethasone in both groups whereas ketorolac which was believed to be useful for the prevention of PCO was used in group 1 only. This means that the second group was left in control. Patients were considered for inclusion in the study if they had a visually significant cataract in the study eye. Exclusion criteria were as follows; glaucoma, uveitis, patients with a history of topical and/or systemic use of steroids or NSAIDs during the previous month, corneal diseases that prevent adequate visualization of the posterior capsule, and allergy to NSAIDs. The eyes were also excluded from the investigation if there were intraoperative unwanted complications such as posterior capsule tears with or without vitreous prolapsus.

A routine thorough medical history was obtained and a complete preoperative ocular examination comprising best-corrected visual acuity, slit-lamp biomicroscopic evaluation, intraocular pressure, and a dilated fundus evaluation were performed. Unilateral or bilateral phacoemulsification with PC-IOL implantation was performed and each eye was accepted as a separate case. All surgeries were performed by the same standardized approach and each eye received the same foldable monofocal hydrophobic acrylic IOL (AcrySof®, Alcon, Fort Worth, USA). All eyes were examined by the same group of ophthalmologists who have many years of experience in treating such patients with cataracts. Preoperatively, pupils were dilated with a combination of topical cyclopentholate 0.5%, tropicamide 0.5%, and phenylephrine 2.5% drops about one hour before the surgery. After two paracenteses were performed on the temporal and nasal corneal periphery, viscoelastic material was injected into the anterior chamber (Healon, Advanced Medical Optics, Santa Ana, CA, USA) and a 2.8-mm clear corneal incision was made with a phaco knife. An anterior continuous curvilinear capsulorhexis was performed with a needle that was completed by the routine capsulorhexis forceps. Hydrodissection and hydrodelamination in some cases were performed, the nucleus was emulsified in the posterior chamber and the remaining lenticular material was aspirated by phaco probe. After a meticulous cortical cleanup was performed, the anterior chamber and the capsular bag were filled with viscoelastic material, and the foldable IOL was implanted in the bag inserted through the main surgical incision. Viscoelastic material was removed from the eye by an irrigation-aspiration probe and the surgery was ended with the injection of intracameral antibiotic.

After the surgery, group 1 eyes received topical ketorolac 0.5% ophthalmic solution four times a day for four weeks whereas group 2 eyes did not receive it. Other regimens were the same for both groups. Therefore, eyes in each group received topical dexamethasone 0.1% drops six times a day for the first week and four times a day for the remaining three weeks. Similarly, both groups received antibiotic drops four to six times a day for a month.

Follow-up examinations were repeated at the same clinic on postoperative first, third, and seventh days, first month, and at six-month intervals thereafter. The patients were instructed to return to the clinic for the assessment of PCO before the next scheduled visit if there was a gradual decrease in visual acuity. The presence of PCO was evaluated after topical mydriatic agents were instilled using 1 drop of both cyclopentholate 1% and tropicamide 0.5% and noted at each visit, if present, and the records were reviewed to determine the frequency and timing of after-cataract formation for each group. The eyes with severe fibrotic posterior capsule opacification that markedly reduces the red reflex and/or Elschnig pearl migration across the visual axis that decreases the visual acuity received Nd:YAG laser posterior capsulotomy.

Statistics

All statistics in the present study were performed using SPSS for Windows (Version 22.0, IBM Corp., Armonk, NY, USA). The results were analyzed using the chi-square test or t-test, as indicated, and final data were expressed as mean±standard error of the mean (SEM) with minimum and maximum values, when available. A p-value less than 0.05 was considered to be significant.

## Results

Surgery was uniform in all patients, comprising a superotemporal clear corneal incision and phacoemulsification with routine posterior capsular polishing and PC-IOL implantation. Two eyes treated with ketorolac and three cases treated without ketorolac were excluded from the study, as they were lost to follow-up. The information about the presence of PCO in patients deceased or lost to follow-up is included for statistical analysis as taken from their last available ophthalmologic examination, provided the follow-up was at least one year. During the study, there were no adverse reactions reported or observed for ketorolac and patients tolerated the topical drops without serious side effects.

A total of 114 eyes of 101 patients (62 men, 39 women) with a mean age of 61.7 years (range, 21 to 85) were enrolled in this randomized study. Eighty-eight cases had unilateral cataracts and 13 patients had bilateral disease. Fifty-four eyes were included in group 1 for the analysis whereas 60 eyes were included in group 2. The mean age for patients with cataract surgery in group 1 (62.8±2.2 years, range, 22-85) and group 2 (60.6±1.7 years, range, 21-79) was similar (p>0.05). The mean postoperative follow-up duration was 24.7±1.3 months for group 1 (range, 15-48) and 25.4±0.7 months for group 2 (range, 16-43). Power analysis for sample size was estimated at 0.9568 for *n*=54, alpha=0.05, degrees of freedom=1, and effect size=0.5.

All patients returned for postoperative examinations as requested for complete ophthalmic examinations with special attention to the presence of PCO. The rate of observed PCO that needed Nd:YAG laser posterior capsulotomy was 2/54 (3.7%) for group 1 and 4/60 (6.6%) for group 2 at two years following phacoemulsification with in-the-bag PC-IOL implantation, and the difference was not significant (p>0.05). The mean month at capsulotomy was 26.5 in group 1 and 24.3 months in group 2 eyes (p>0.05).

## Discussion

Based on the findings from various in vivo and in vitro investigations in animals as well as in humans that NSAIDs can lessen the formation of PCO following cataract surgery [12,13], some new non-invasive clinical approaches to reduce after-cataract formation in humans are potentially important because complications from YAG capsulotomy after cataract surgery may result in significant loss of vision from cystoid macular edema, retinal breaks, and detachments with possible IOL damage or subluxation in some cases [1-7].

Flach and Dolan [14] demonstrated less frequent PCO following treatment with ketorolac ophthalmic solutions when compared with diclofenac drops, though the difference was not significant three years after cataract surgery and implantation of a foldable silicone IOL. However, because the authors tested two topical ocular NSAIDs, it is difficult to evaluate the exact effect of ketorolac or diclofenac for the prevention of PCO formation in adults as there is no control group that did not use this agent. Indeed, the authors agreed that further studies in this area were indicated. Similarly, the incidence of PCO was found to be similar in eyes treated either with diclofenac or betamethasone drops [15]. Although Zaczek et al. [16] compared diclofenac, dexamethasone, and a placebo in the immediate period after phacoemulsification and IOL implantation, this study did not include ketorolac again. Therefore, it is not still clear whether postoperative use of steroids and NSAIDs has an effect on lens epithelial proliferation and the formation of PCO and it is difficult to make a definitive conclusion about the possible inhibitory effect of ketorolac ophthalmic solutions on the incidence of PCO following cataract removal in adult patients. It is conceivable to compare the incidence of postoperative PCO following treatment between combined dexamethasone plus ketorolac versus dexamethasone alone.

In light of these data, we aimed in the current study to test this hypothesis in a randomized clinical trial and planned a two-year analysis to find out whether steroidal with or without non-steroidal treatment reduces the formation of PCO following cataract surgery. The overall incidence of PCO in a total of two eyes in group 1 treated with topical dexamethasone plus ketorolac eye drops during the early postoperative period was similar to that found in a total of four eyes in group 2 treated with dexamethasone alone, indicating that ketorolac had no additional influence on the formation of PCO two years after the surgery. Our findings agree with those articles that demonstrated a similar incidence of PCO after phacoemulsification following postoperative treatment with diclofenac, with dexamethasone, or with ketorolac eye drops when they were given alone for each case. In other words, we demonstrated further that combined applications of steroidal and non-steroidal eye drops do also not reduce the incidence of PCO at two years postoperatively. All of these six eyes with severe PCO in both groups had to have Nd:YAG laser capsulotomy two years following the surgery.

Growing evidence suggests that implanted IOL designs, materials, and haptic configuration may influence PCO rates, though the results are controversial [4,17-19]. Indeed, hydrophobic material, smooth optic surface, and sharp-edged IOLs play a key role in PCO development and are likely to be associated with less PCO formation than round-edged IOLs, with less Nd:YAG capsulotomy [5,20,21]. Similarly, Nd:YAG rates were reported to be significantly lower with AcrySof IOLs when compared with Tecnis IOLs [3]. On the other hand, both the PCO value and the Nd:YAG capsulotomy rates were found to be higher in the hydrophobic acrylic IOLs group than in the silicone IOLs group at long-term use after implantation [4]. Therefore, to minimize the possible effects of these factors, we chose one type of IOL for each group [20,21]. In addition, every attempt was also performed to reduce the incidence of after-cataract formation by performing atraumatic surgery with a continuous curvilinear capsulorhexis and a complete residual cortical cleanup after surgery with meticulous posterior capsule cleaning and “in-the-bag" implantation of the IOL in both groups [22].

This study has three limitations. First, group 1 eyes were treated with topical ketorolac four times daily only for four weeks postoperatively. Therefore, a longer duration of treatment or frequent doses may provide different findings as we demonstrated in our pediatric cataract cases [10]. In addition, although a two-year follow-up in the present study seems to be sufficient to make clinically relevant conclusions in adult cases with cataract surgery, a longer follow-up time may reveal different results. Second, a simple observation during slit-lamp biomicroscopic examinations was used as a method of determining PCO as previously was used in many investigations. However, more sophisticated methods, namely high-resolution digital retroillumination images taken with a Scheimpflug camera, being used to grade the amount of PCO by measuring, grading, and photographing would yield more consistent results. Third, although the power analysis revealed a favorable result, further studies with a larger sample size may reveal different findings because there was a favorable trend toward the ketorolac group in this research, though it was not statistically significant.

## Conclusions

More and more investigations are being performed for the prevention of PCO, which is the most common postoperative complication following cataract surgery in the world representing about half of the cases, most of which require Nd:YAG laser capsulotomy. Some modifications in surgical techniques were suggested such as meticulous cortical cleanup, sharp-edged optic design, and various pharmacological compositions that have been reported to reduce the incidence of PCO formation. However, this complication is seen frequently.

Although our team and some other clinicians advocate using diclofenac or ketorolac eye drops following phacoemulsification to prevent PCO, especially after pediatric cataract surgeries, the present clinical trial in adults did not show any difference for the combination of topical dexamethasone plus ketorolac ophthalmic solutions when compared with dexamethasone eye drops alone, though it may be effective for the control of intraocular inflammation. An interesting future area of study might be the use of intracameral NSAIDs at the time of surgery. The adverse effects of long-term ketorolac use must be taken into account. Therefore, we should continue to search some novel drugs or ophthalmic solutions which are used pre and/or postoperatively to prevent the formation of PCO after cataract surgery.
